# Ipsilateral occult hernias during endoscopic groin hernia repair

**DOI:** 10.4103/0972-9941.41946

**Published:** 2008

**Authors:** Mayank Jain, Shashi Khanna, Bimalendu Sen, Om Tantia

**Affiliations:** Department of Minimal Access Surgery, ILS Multispeciality Clinic, DD-6, Sector – I, Salt Lake City, Kolkata - 700 064, India

**Keywords:** Multiple groin hernia, occult hernia, trans-abdominal pre-peritoneal, totally extra-peritoneal

## Abstract

Endoscopic repair of groin hernias allows the surgeon to have a complete view of the groin and pelvis to diagnose occult hernias both ipsilaterally and contralaterally. These occult hernias can then be treated simultaneously and may reduce the incidence of recurrence and persistent symptoms. The authors present four unusual cases where occult hernias were found ipsilaterally during an endoscopic repair. All these occult hernias were treated along with the clinically diagnosed hernia at the same surgery with excellent results and no post-operative morbidity.

## INTRODUCTION

With the introduction of endoscopic repair of groin hernia in 1991 by Ger[1] there had been tremendous interest in the procedure by the surgeons all around the world. In addition to its well known benefits of decreased postoperative pain and shorter recovery time, laparoscopic repair has a major advantage of allowing the surgeon to diagnose occult ipsilateral and contra-lateral groin hernias.[2–4]

If these incipient hernias were left untreated, the patient would either have continued to be symptomatic or would report back to the surgeon with recurrence.[4]

We report a case series of four such cases where ipsilateral occult hernias were diagnosed while doing endoscopic repair of groin hernias and were treated simultaneously with excellent results.

## CASE REPORT

Four cases have been diagnosed with occult hernias while doing endoscopic groin hernia repair over the last one year (from Sept 06 to Aug 07) at our institute. The demographic data, clinical diagnosis, per-operative findings and follow-up has been mentioned in Table 1. All these patients had complains of swelling and intermittent pain on the diseased groin. None of the patient had history suggestive of irreducibility, obstruction or strangulation. The patients were averagely built and general examination was unremarkable. One of the patient had history of hypertension which was controlled on medications and other had history of ischemic heart disease but was otherwise asymptomatic. His Echocardiogram showed an ejection fraction of 55% and was categorised as ASA grade II.

**Table 1:** Demographic data, clinical diagnosis, per-operative findings and follow-up of the patients. Occult groin hernias are shown in bold characters in per-operative findings.

S/N	Age	Sex	Clinical	Past history diagnosis	Per-op findings	Procedure done	Operative	H/Stay time (mins)	F/U (months)
1	64	M	Bilateral inguinal hernia (both indirect)	Ischemic Heart Disease Lt. Tibial nailing	B/L indirect inguinal hernia with Lt femoral + Rt obturator hernia	Lap Bilateral TEP	80	2 days	6
2	70	M	Rt inguinal herina (direct)	Hypertension	Rt direct inguinal hernia with Rt femoral + obturator hernia	Lap TAPP	60	1 day	6
3	48	F	Lt inguinal herina (indirect)	Tubal ligation Laparoscopic Assisted Vaginal Hysterectomy + Bilateral Salphingo-Oophorectomy	Lt indirect inguinal hernia with Lt obturator + Lt femoral hernia	Lap TAPP	60	2 days	3
4	54	M	Rt inguinal hernia (direct)	NS	Rt direct inguina I hernia with Rt femoral hernia	Lap TAPP	40	1 day	10

TEP - Totally extraperitoneal repair, TAPP - Transabdominal pre-peritoneal repair

**Figure 1:**
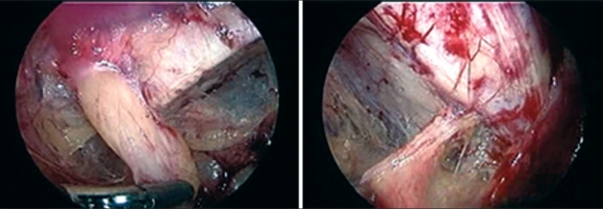
Endoscopic view of groin of patient 1, showing right femoral and left obturator hernia

**Figure 2:**
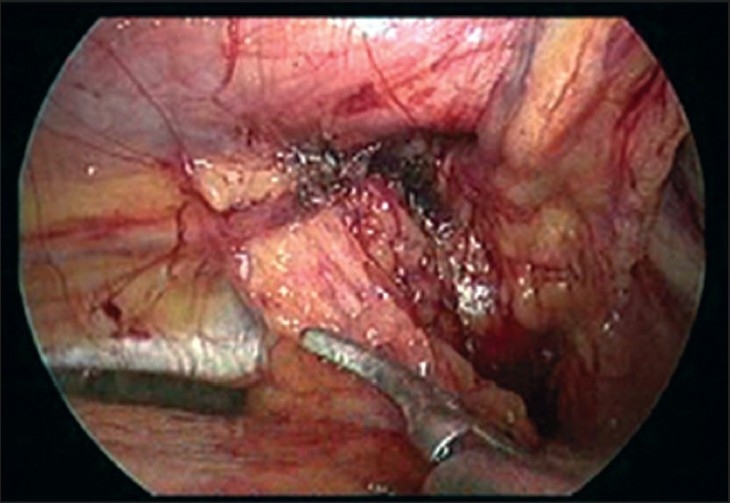
Endoscopic view of groin of patient 2, showing right femoral hernia and right direct inguinal hernial defect

All patients were operated under general anesthesia. A standard three port technique was used with midline 10 mm optical port at umbilicus and right and left side 5 mm working ports at right and left mid-clavicular line respectively slightly below the umbilicus. In two cases the procedure was done by trans-abdominal pre-peritoneal (TAPP) technique while in other two by totally extra-peritoneal (TEP) technique. The standard technique of TAPP / TEP procedure was adopted. Decision to undertake TAPP or TEP was randomised since we are also doing a comparative study of TAPP *vs* TEP repaired hernias. Both groins were visualised during TAPP technique while during TEP only the symptomatic groin was explored. Endoscopic view of the groin of patient 1 and patient 2 after complete dissection is shown in Figure 1 and 2 respectively.

While doing a TAPP repair, 10 mm optical port was first made at the umbilicus by closed technique. Both the groins was inspected and right and left hand working ports were inserted under vision taking care to avoid injury to inferior epigastric vessels. Ipsilateral peritoneal flap was raised with its lateral extent being 1 cm above and medial to anterior superior iliac spine (ASIS) and medial extent being just lateral to medial umbilical ligament. Further medial dissection was continued behind the medial umbilical ligament and os pubis was identified. Complete parietalisation of cord structures was done and lateral dissection was continued after lysis of fascia of Dulucq near the internal inguinal ring up to close to the anterior superior iliac spine. Direct / indirect inguinal hernia or femoral or obturator hernia were identified, dissected and reduced during the procedure.

A polypropylene mesh of 15 × 12 cm (Prolene - Ethicon, Somerville, New Jersey) was placed and secured in position by tacks (Protack, Autosuture, Tyco Healthcare, United States Surgical). Two tacks were used at pubic bone and Cooper's ligament to fix the mesh adequatey so that it adequately covered the obturator, inguinal and femoral orifices. Peritoneal flap was then re-sutured with Vicryl 2 - 0.

While doing the TEP technique, the same three ports was used. Hasson's canula was used for the camera port and the initial pre-peritoneal space was created by to and fro movements of 10 mm, 0 degree telescope under vision and the first important landmark identified was os pubis. Lateral dissection was then done similarly and after adequate working space was created, right and left hand working ports were put under vision and the telescope was changed to 10 mm, 30 degree. Principles and extent of further dissection was same as that of TAPP. 15 × 12 cm mesh was properly placed and secured. In one case where the diagnosis was bilateral groin hernia, other side was also dissected and all hernias were reduced.

All the patients were ambulatory within six hours of surgery and were allowed liquid diet. Soft diet was started the next day and patients were discharged. One patient had a stay of two days (< 48 hours) because of slight pain which he was complaining on day 1. Mean operative time was one hour.

Patients were followed on day seven and subsequently at one month and six months. Postoperative period was uneventful and all were symptom free at their last follow-up. None of the patients had seroma, chronic pain or recurrence after surgery.

## DISCUSSION

Diagnosis of unsuspected groin and pelvic hernias is not uncommon while doing endoscopic repair of groin hernias. Most of these are either femoral or obtruator hernias and have been defined endoscopically by the presence of herniating pre-peritoneal tissue within the canal.[2] One of the reason for failure of clinical examination to diagnose these femoral and obturator hernias may be their rarity. While femoral hernia may make up just about 5% of all patients with groin hernia,[5] obturator hernia represents less than 1% of all hernias.[6]

Besides these ipsilateral occult hernias unsuspected contralateral hernias may also be diagnosed by endoscopic hernia surgery. The incidence of occult contra lateral hernias has been mentioned between 10-20%.[7] A study by Crawfard *et al*, has shown that of the 73 patients diagnosed with unilateral hernias, 37 patients (50%) had bilateral hernia on diagnostic laparoscopy. To add on this 13 patients (18%) had either a different type of inguinal hernia (7 patients) or a different hernia all-together (femoral hernia in 6 patients). Thus the diagnosis was correct in only 32% of patients.[2]

Another study by Koehler concluded that patients believe to have unilateral inguinal hernia have occult contra lateral hernias in 13% cases on diagnostic laparoscopy. The actual bilateral hernia incidence was 25%, with 37% false positive rate for preoperatively diagnosed bilateral hernias.[3]

Ekberg *et al*, studied 550 patients and concluded that femoral hernia may be present together with other hernias in ipsilateral and contra-lateral groin.[8] In their later series on ipsilateral multiple groin hernias in 314 patients, 71 patients (23%) had multiple hernias. Ipsilateral multiple hernias were found in 18 patients (6%). Multiple hernias were present ipsilaterally in 6% patients with indirect hernia, 12% patients with direct hernia, 21% patients with femoral hernia and 23% patients with obturator hernia.[4]

A misdiagnosed or overlooked hernia may account for some of the so called recurrence or persisting symptoms after surgery.[4] As such it may be necessary to properly identify the hernia and also to look for occult ones and treat them simultaneously.

Endoscopic repair allows diagnosis of unsuspected groin and pelvic hernias which can also be repaired during the same surgery with no added morbidity and thereby may reduce the incidence of post-op recurrence and persistent symptoms due to these occult hernias.
